# Efficacy of Single Injection of Platelet-Rich Plasma in Shoulder Impingement Syndrome

**DOI:** 10.7759/cureus.25727

**Published:** 2022-06-07

**Authors:** Shivam Saurav, Aditya N Aggarwal, Pratyush Shahi, Sushil Kamal, Kuldeep Bansal, Shubham Singla

**Affiliations:** 1 Orthopaedics, University College of Medical Sciences, New Delhi, IND

**Keywords:** visual analog scale (vas), range of motion, single injection, shoulder impingement, platelet-rich plasma

## Abstract

Introduction

To analyze the change in Visual Analog Scale (VAS), QuickDASH score, and the range of motion at the shoulder joint following a single injection of platelet-rich plasma (PRP) in shoulder impingement syndrome.

Methods

Twenty patients (21 shoulders) of either sex above the age of 18 years with a clinical diagnosis of shoulder impingement having a positive shoulder impingement test (positive Hawkins-Kennedy impingement test and/or positive Neer's impingement sign), ultrasonographic confirmation of shoulder impingement, and a failure to respond to standard non-operative methods for a minimum period of four weeks were included in this prospective interventional study. PRP was injected at the proposed site. At three months after the injection, the changes in the VAS, QuickDASH score, and the range of motion at the shoulder joint were analyzed.

Results

There were significant changes in the VAS, QuickDASH score, and range of motion at the shoulder joint following a single injection of PRP.

Conclusions

Platelet-rich plasma (PRP) injection results in a significant decrease in pain and improvement in the range of motion and an overall excellent functional outcome in shoulder impingement syndrome. However, future studies with a bigger sample size and longer follow-up are needed.

## Introduction

Shoulder disorders are one of the most common musculoskeletal disorders in general practice [1]. In regular practice, the incidence of shoulder disorders is approximately 12-25 per 1,000 cases seen per year [2]. There are many differential diagnoses of shoulder pain like shoulder impingement syndrome, calcific tendinitis, and adhesive capsulitis, out of which shoulder impingement syndrome accounts for 44% to 65% [3].

There are multiple treatment modalities of shoulder impingement syndrome which can be classified as operative and non-operative. Non-operative therapy includes physical therapy, non-steroidal anti-inflammatory drugs (NSAIDs), and injections of corticosteroid, platelet-rich plasma (PRP), and hyaluronic acid. Most of the patients respond to non-operative therapy; however, there is no consensus on the duration of conservative treatment for shoulder impingement syndrome [4].

Platelet-rich plasma (PRP) is a bioactive component of whole blood with an elevated concentration of platelets. It has a high concentration of growth factors that act on the degenerative tissue leading to healing by collagen synthesis, revascularization, and proliferation of stem cells into tissue-specific cells [5]. It has been successfully used in the treatment of various degenerative conditions. To the best of our knowledge, there are very few published studies, none being from the Indian subcontinent, that show the effect of PRP injection in shoulder impingement syndrome. Hence this study was proposed.

## Materials and methods

Twenty (21 shoulders) patients of either sex above the age of 18 years with a clinical diagnosis of shoulder impingement having a positive shoulder impingement test (positive Hawkins-Kennedy impingement test and/or positive Neer’s impingement sign), ultrasonographic confirmation of shoulder impingement, and a failure to respond to standard non-operative methods (exercise and tramadol) for a minimum period of four weeks were included in this prospective interventional study. Patients with a previous history of diabetes mellitus, anemia, septicemia, complete rotator cuff tear, previous steroid injection to the shoulder, and previous shoulder surgery were excluded from the study. Ethical clearance was given by the Institutional Ethics Committee, and informed consent was obtained from each patient.

Before the injection of platelet-rich plasma, all the patients were evaluated for pain according to the Visual Analog Scale (VAS) [6]. The range of motion of the shoulder was recorded. Flexion, abduction, and external rotation were recorded with the help of a goniometer, while for recording internal rotation, the patients were asked to touch their back from down above. The highest point where their thumb could reach was recorded. The QuickDASH questionnaire was filled by every patient [7].

The double-spin method was used for the preparation of PRP. The patient’s blood (7.5 ml) was drawn by venipuncture and collected in an anticoagulant-citrate-dextrose (ACD) tube containing 1.5 ml of the anticoagulant. An ethylenediaminetetraacetic acid (EDTA) sample was also collected at the same time for determining the baseline counts of the patient. The ACD tube was centrifuged at 160 g (1,300 revolutions per minute (rpm)) for 10 minutes at 20°C (soft spin). The PRP formed was aspirated into a sterile tube. The sterile tube containing PRP was centrifuged at 400 g (2,000 rpm) for 10 minutes at 20°C. The supernatant PRP was roughly reduced to 1/4th of the volume, and the platelet pellets were resuspended resulting in the production of leukocyte poor platelet concentrate. Calcium gluconate (0.2 ml), which acts as an activator, was added to the syringe containing PRP just before the application of PRP in the patient.

The shoulder to be injected with PRP was cleaned with spirit, and a sterile drape was placed at the proposed site. The subacromial space was accessed through the lateral approach. The injection needle was inserted just inferior to the mid-lateral aspect of the acromion with the needle being angled slightly cephalad. It was then passed through the deltoid and directed medially and slightly anteriorly into the subacromial-subdeltoid bursa. Care was taken to avoid injecting directly into the tendon of the rotator cuff.

Post-injection, the patients were advised to not take any NSAIDs and to avoid high-impact activity for at least a week. Patients were continued on pain-free gentle shoulder pendulum exercises followed by passive and active range of motion exercises. This was followed by rotator cuff strengthening exercises as permitted by pain. They also explained about the possibility of increased discomfort in the first week after PRP injection due to local soft tissue injury.

The patients were followed up at six weeks. A reduction in symptoms and any other complaints was noted. The patients were then reviewed 12 weeks after the injection. The VAS score, QuickDASH score, and range of motion of the shoulder were analyzed in this visit. Any other complication was recorded. Descriptive statistics summarizing the patient’s characteristics were analyzed. Wilcoxon signed-rank test was used to compare the VAS scores before injecting PRP and after 12 weeks of PRP injection. The QuickDASH score and change in range of motion of the shoulder were also analyzed.

## Results

The mean age of the patients was 46.1 years. Out of the 20 patients, nine were males and 11 were females. Nineteen patients had unilateral shoulder impingement, and one patient had bilateral impingement. The mean pre-injection VAS was 5.1 ± 1.39, while the mean VAS recorded after three months of the injection was 3.3 ± 1.72. The P-value calculated by Wilcoxon signed-rank test was less than 0.001 and hence significant. The reduction in VAS for pain is summarized in Table 1.

**Table 1:** Outcome measures in terms of reduction in Visual Analog Scale (VAS), change in forward flexion, abduction, external rotation, and internal rotation after three months of the platelet-rich plasma injection.

Reduction in Visual Analog Scale (VAS)	No. of shoulders	Percentage
≥ 50%	10	47.6%
≥ 20 % to 50 %	6	28.5%
< 20 %	5	23.8%
Change in forward flexion		
> 30 degrees	7	33.33%
10 to 30 degrees	13	61.9%
< 10 degrees	1	4.7%
Change in abduction		
> 25 degrees	7	33.33%
10 to 25 degrees	13	61.9%
< 10 degrees	1	4.7%
Change in external rotation		
< 5 degrees	13	61.92%
5 to 10 degrees	4	19.04%
> 10 degrees	4	19.04%
Change in internal rotation		
No improvement	5	23.8%
1 to 3 vertebra	7	33.3%
≥ 4 vertebra	7	33.3%

QuickDASH score could not be recorded in one patient who was having an amputation of the second, third, and fourth fingers of the ipsilateral hand. The mean pre-injection QuickDASH score in the remaining 19 patients was 46.5 ± 12.9, and after three months of the injection, it was 29.9 ± 14.6. The reduction in mean QuickDASH following PRP injection was 35.6%. The mean pre-injection forward flexion was 128.57 degrees ± 30.42 degrees, and after three months of the injection, it was 148.57 degrees ± 27.02 degrees. There was a mean increase of 20 degrees. The change is summarized in Table 1.

The mean pre-injection abduction at the shoulder joint was 112.62 degrees ± 42.47 degrees, and after three months of the injection, it was 135.29 degrees ± 40.01 degrees. There was a mean increase of 22.6 degrees. The change is summarized in Table 1. The mean pre-injection external rotation was 65.71 degrees ± 17.55 degrees, and after three months of the injection, it was 68.57 degrees ± 16.51 degrees. The change is summarized in Table 1.

The internal rotation was quantified in terms of improvement in vertebral level using Apley's scratch test in which the patients were asked to touch their back from down above. The improvement in the internal rotation is summarized in Table 1. One patient, whose pre-injection internal rotation was restricted to the gluteal fold, could move her thumb to the sacroiliac joint after three months of the injection. Another patient, whose pre-injection internal rotation was restricted to the sacroiliac joint, could move her thumb to the third lumbar vertebra.

## Discussion

Shoulder impingement results from an “inflammation and degeneration of the anatomical structures in the region of the subacromial space” [8]. Both intrinsic and extrinsic factors are held responsible for shoulder impingement syndrome. Extrinsic factors include extratendinous causes such as stress-induced spurs on the underside of the acromion, acromioclavicular joint osteophytes, pathological os acromiale, and dysfunction of the scapulothoracic and glenohumeral joints. Intrinsic factors include rotator tendon dysfunction due to age-related changes, diabetes mellitus, and steroid intake [4]. The patients present with pain and restricted movements of the shoulder joint. On examination, a positive Hawkins-Kennedy test and Neer’s impingement sign can be seen [9]. Ultrasound has been widely used for the diagnosis of shoulder impingement syndrome. The sensitivity and specificity of ultrasound (USG) for detecting rotator cuff disorders are comparable to those of MRI [10].

Both operative and non-operative treatment modalities have been described for the treatment of shoulder impingement syndrome. Operative treatment should be offered to the patients not responding to a fair trial of conservative treatment. Although PRP has been successfully used in the treatment of various degenerative conditions, there have been very few studies that evaluate its role in shoulder impingement syndrome.

In this study, the changes in VAS for pain, QuickDASH score, and range of motion at the shoulder after PRP injection were all statistically significant. The previous studies had noted similar findings [11-15]. PRP was prepared by the double-spin method in our study. Citrate dextrose was used as an anticoagulant, and calcium gluconate was used as an activator. The technique of PRP and the anticoagulant and activator used in the previous studies have been summarized in Table 2 [11-18].

**Table 2:** Methods of platelet-rich plasma preparation, and anticoagulants and activating agents used in the previous studies.

Studies	Platelet-rich plasma preparation method	Anticoagulant used	Activating agent
Rha et al. (2013) [16]	Single spin	Citrate dextrose	Calcium chloride
Kesikburun et al. (2013) [14]	Single spin	Citrate dextrose	None
Scarpone et al. (2013) [13]	Single spin	Citrate dextrose	None
Ilhanli et al. (2015) [15]	Single spin	Citrate dextrose	Calcium chloride
Wesner et al. (2016) [18]	Single spin	Citrate dextrose	None
Shams et al. (2016) [17]	Single spin	Citrate dextrose	None
Say et al. (2014) [12]	Single spin	Citrate dextrose	Calcium chloride
Nejati et al. (2017) [11]	Double spin	Citrate dextrose	None
Current study	Double spin	Citrate dextrose	Calcium gluconate

All the previous studies had used citrate dextrose as an anticoagulant solution. Some studies did not use an activating agent. When an activator is not used, PRP gets activated by coming in contact with the tissue collagen [5]. We used calcium gluconate as the activating agent. Considering the concern of migration of PRP away from the intended region, it has been hypothesized that the activation of PRP before the injection to the injured tissue leads to an improved concentration of growth factors at the site of injection [19]. However, Scherer et al. examined the effects of platelet activation on wound healing in vivo and in vitro and found that the wounds treated with non-activated PRP healed more quickly [20]. Growth factors might have a more optimal effect when present on-demand, rather than being released immediately at the time of injection [21].

In various studies, multiple injections of PRP were given. Nejati et al. gave two injections of PRP at an interval of one month, Ilhanli et al. gave three injections of PRP at an interval of one week, and Rha et al. gave two injections of PRP at an interval of four weeks [11,15,16]. In the current study, a single injection of PRP was given. In the previous studies, the volume of PRP injected ranged from 2 ml to 6 ml. In the current study, 2 ml of PRP was injected. This was the volume of PRP produced after centrifugation.

In various previous studies, PRP was injected under ultrasonographic guidance [11,13,14,18]. We used the lateral approach to the shoulder for the injection of PRP. Although it can be accepted that the ultrasonographic guidance aids in more accurate placement of the needle and this may be perceived as a shortcoming of our study, a randomized controlled trial (RCT) done by Bhayana et al. showed no advantage of ultrasonographic guidance over the landmark approach when corticosteroid was injected for shoulder impingement syndrome [22]. Bloom et al. also were unable to establish an advantage in terms of pain, function, and shoulder range of motion of ultrasound-guided glucocorticoid injection over the landmark approach [23]. In various previous studies, local anesthesia was used along with the PRP injection [12,13]. A few studies have shown a decrease in the proliferation of tenocytes and cell viability leading to less positive results when PRP is combined with local anesthesia [24]. In the current study, local anesthesia was not used.

Limitations

Our study's limitations include a relatively small sample, the lack of a control group treated conservatively or with placebo/corticosteroid injections, a shorter follow-up period, and the use of a landmark approach rather than an ultrasonographic needle insertion method.

## Conclusions

Shoulder impingement is a common cause of pain and dysfunction in young adults. Various non-operative treatment modalities have been described in the literature for its management. The role of platelet-rich plasma for the treatment of impingement syndrome has been a matter of debate. According to our study, platelet-rich plasma (PRP) injection results in a significant decrease in pain and improvement in the range of motion and an overall excellent functional outcome in shoulder impingement syndrome. However, future studies with a bigger sample size and longer follow-up are needed.

## References

[1] Urwin M, Symmons D, Allison T (1998). Estimating the burden of musculoskeletal disorders in the community: the comparative prevalence of symptoms at different anatomical sites, and the relation to social deprivation. Ann Rheum Dis.

[2] van der Windt DA, Koes BW, de Jong BA, Bouter LM (1995). Shoulder disorders in general practice: incidence, patient characteristics, and management. Ann Rheum Dis.

[3] Ostör AJ, Richards CA, Prevost AT, Speed CA, Hazleman BL (2005). Diagnosis and relation to general health of shoulder disorders presenting to primary care. Rheumatology (Oxford).

[4] Singh B, Bakti N, Gulihar A (2017). Current concepts in the diagnosis and treatment of shoulder impingement. Indian J Orthop.

[5] Etulain J (2018). Platelets in wound healing and regenerative medicine. Platelets.

[6] Carlsson AM (1983). Assessment of chronic pain. I. Aspects of the reliability and validity of the visual analogue scale. Pain.

[7] Kennedy CA, Beaton DE, Smith P (2013). Measurement properties of the QuickDASH (disabilities of the arm, shoulder and hand) outcome measure and cross-cultural adaptations of the QuickDASH: a systematic review. Qual Life Res.

[8] Michener LA, Walsworth MK, Doukas WC, Murphy KP (2009). Reliability and diagnostic accuracy of 5 physical examination tests and combination of tests for subacromial impingement. Arch Phys Med Rehabil.

[9] Phillips N (2014). Tests for diagnosing subacromial impingement syndrome and rotator cuff disease. Shoulder Elbow.

[10] Elmorsy A, Keightley A, Flannery M (2017). Accuracy of ultrasonography (US) and magnetic resonance imaging (MRI) in detection of rotator cuff tears in district general hospital. Pol J Radiol.

[11] Nejati P, Ghahremaninia A, Naderi F, Gharibzadeh S, Mazaherinezhad A (2017). Treatment of subacromial impingement syndrome: platelet-rich plasma or exercise therapy? A randomized controlled trial. Orthop J Sports Med.

[12] Say F, Gürler D, İnkaya E, Bülbül M (2014). Comparison of platelet-rich plasma and steroid injection in the treatment of plantar fasciitis. Acta Orthop Traumatol Turc.

[13] Scarpone M, Rabago D, Snell E (2013). Effectiveness of platelet-rich plasma injection for rotator cuff tendinopathy: a prospective open-label study. Glob Adv Health Med.

[14] Kesikburun S, Tan AK, Yılmaz B, Yaşar E, Yazıcıoğlu K (2013). Platelet-rich plasma injections in the treatment of chronic rotator cuff tendinopathy: a randomized controlled trial with 1-year follow-up. Am J Sports Med.

[15] Ilhanli I, Guder N, Gul M (2015). Platelet-rich plasma treatment with physical therapy in chronic partial supraspinatus tears. Iran Red Crescent Med J.

[16] Rha DW, Park GY, Kim YK, Kim MT, Lee SC (2013). Comparison of the therapeutic effects of ultrasound-guided platelet-rich plasma injection and dry needling in rotator cuff disease: a randomized controlled trial. Clin Rehabil.

[17] Shams A, El-Sayed M, Gamal O, Ewes W (2016). Subacromial injection of autologous platelet-rich plasma versus corticosteroid for the treatment of symptomatic partial rotator cuff tears. Eur J Orthop Surg Traumatol.

[18] Wesner M, Defreitas T, Bredy H, Pothier L, Qin Z, McKillop AB, Gross DP (2016). A pilot study evaluating the effectiveness of platelet-rich plasma therapy for treating degenerative tendinopathies: a randomized control trial with synchronous observational cohort. PLoS One.

[19] Fufa D, Shealy B, Jacobson M, Kevy S, Murray MM (2008). Activation of platelet-rich plasma using soluble type I collagen. J Oral Maxillofac Surg.

[20] Scherer SS, Tobalem M, Vigato E (2012). Nonactivated versus thrombin-activated platelets on wound healing and fibroblast-to-myofibroblast differentiation in vivo and in vitro. Plast Reconstr Surg.

[21] Mautner K, Kneer L (2014). Treatment of tendinopathies with platelet-rich plasma. Phys Med Rehabil Clin N Am.

[22] Bhayana H, Mishra P, Tandon A, Pankaj A, Pandey R, Malhotra R (2018). Ultrasound guided versus landmark guided corticosteroid injection in patients with rotator cuff syndrome: randomised controlled trial. J Clin Orthop Trauma.

[23] Bloom JE, Rischin A, Johnston RV, Buchbinder R (2012). Image-guided versus blind glucocorticoid injection for shoulder pain. Cochrane Database Syst Rev.

[24] Carofino B, Chowaniec DM, McCarthy MB (2012). Corticosteroids and local anesthetics decrease positive effects of platelet-rich plasma: an in vitro study on human tendon cells. Arthrosc J Arthrosc Relat Surg.

